# Reconstructing History: Scale Analysis Reveals Long‐Term Changes in Age‐Related Growth of a Coregonid Fish

**DOI:** 10.1002/ece3.71884

**Published:** 2025-07-30

**Authors:** Christian Vogelmann, Maxim Teichert, Michael Schubert, Niels Dingemanse, Herwig Stibor

**Affiliations:** ^1^ Faculty of Biology Department II, Aquatic Ecology Ludwig‐Maximilians‐Universität München Martinsried Germany; ^2^ Bavarian State Research Center for Agriculture (LfL), Institute for Fisheries Starnberg Germany; ^3^ Austrian Federal Agency for Water Management Institute for Aquatic Ecology and Fisheries Management, Scharfling Mondsee Austria; ^4^ Faculty of Biology Department II, Behavioural Ecology Ludwig‐Maximilians‐Universität München Martinsried Germany

**Keywords:** fisheries induced evolution, maturation shifts, reproductive allocation, whitefish

## Abstract

Animal growth is shaped by a complex interplay of environmental conditions and intrinsic life‐history trade‐offs, yet long‐term datasets allowing the reconstruction of individual growth histories in natural populations remain rare. Here, we use scale analysis to reconstruct age‐specific growth histories of coregonid fish (*Coregonus* sp.) from Lake Starnberg, Germany, over a 22‐year period, evaluating the roles of lake phosphorus concentrations and spring temperatures as potential drivers. Linear mixed‐effects models revealed age‐specific changes in scale growth over time: while growth remained stable or increased slightly in younger age classes (ages 1 and 2), growth significantly declined over time in mature fish (age 3). During the observed period, phosphorus concentrations decreased, indicating reduced nutrient availability, whereas spring temperatures showed no significant long‐term trend. Our analyses further indicated significant interactions between temperature and age class, suggesting that temperature effects on growth may have varied by age, even though there was no overall change in temperature during the monitored time period. The observed decline in growth among mature individuals aligns with predictions from life‐history theory, reflecting a potential allocation shift from somatic growth to reproductive investment following maturation. This study provides rare empirical evidence from a natural fish population that long‐term environmental changes interact with intrinsic life‐history strategies, resulting in clear age‐specific patterns of growth variation.

## Graphical abstract

Model fit for change in scale radius length over time by age class, based on linear mixed effect models for (i) radius length as a function of year, age class, and subsequent interaction (dashed lines), and (ii) radius length as a function of year, temperature, phosphorus, and age class, as well as interactions between temperature and phosphorus with age class.

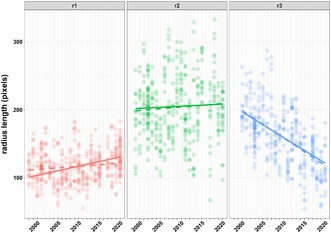

## 1 Introduction

Animal growth is affected by multiple different factors, including external determinants such as food availability (Bertalanffy 1957; West et al. 2001) and temperature (Brown et al. 2004; Gillooly et al. 2001), as well as intrinsic ones like life‐history strategy (Hofmann et al. 1999) and age (West et al. 2001). While sufficient nutrition is an obvious prerequisite for growth (Bertalanffy 1957; Gillooly et al. 2001), the effects of temperature, life‐history strategy, and age are more complex and interdependent. Several well‐accepted ecological rules predict decreasing size with increasing temperatures for most organisms (i.e., Bergmann's rule, Jame's rule, Temperature‐Size rule) (Bergmann 1848; James 1970; Atkinson 1994), but the effect of temperature may differ by life‐history stage and body size (Forster and Hirst 2012). Growth also generally decreases with age (West et al. 2001), but the exact trajectory depends on the onset of maturity and the resulting trade‐off between somatic and reproductive growth (Roff et al. 2006). In turn, size and age at maturity are affected by environmental growth conditions (Plaistow et al. 2004) and may further be subject to selection pressure by size‐specific predation (Abrams and Rowe 1996; Reznick et al. 2008). Gaining a deeper understanding of the processes that influence growth patterns is crucial for effectively managing economically significant animal resources like game, marine life, and freshwater fish. These populations are often heavily impacted by human activities, which can greatly alter growth patterns and population dynamics, including the risk of extinction or reduction of sustainable harvest yield.

In many parts of Europe, North America, and Asia, coregonids are an important economic target species for freshwater fisheries and often the main source of income for local fishermen (Anneville et al. 2009; Baer et al. 2017). Over the last decade, various coregonid populations in European lakes have exhibited a reduction in population size and growth (Müller et al. 2007), which is mainly thought to be related to decreases in lake productivity (Numann 1972; Kirchhofer 1995; Mueller and Bia 1998). While coregonid diet quality and quantity have been shown to be dependent on nutrient availability (Svarvar and Müller 1982; Müller et al. 2007; Jochimsen et al. 2013) as well as temperature effects (Helland et al. 2006; Ohlberger et al. 2011; Cross et al. 2014), such declines have already resulted in critically low abundances or even collapse of local coregonid populations (Sarvala et al. 1988; Perrier et al. 2012). However, current understanding of the drivers of long‐term change in coregonid growth patterns is lacking. Long‐term fisheries data are mainly based on seasonal/annual fish landings. These data do not readily facilitate the tracking of individual fish growth patterns, which is essential for gaining a deeper understanding of fish growth dynamics.

Analyses of various hard structures in fish, such as operculum, vertebral bones, otoliths, fin rays, and scales allow for determining age, condition, and stress, as well as the growth history of fish (Klumb et al. 1999; Cheung et al. 2007; Muir et al. 2008). Especially for short‐lived and fast‐growing fish, scales are a suitable choice for such analyses (Chilton and Beamish 1982; Fisher and Pearcy 1990; Cheung et al. 2007) and many studies show that age determination in coregonids based on scales has been successful (van Oosten 1923; Van Oosten and Hile 1949; Chilton and Beamish 1982; Wilson and Pitcher 1984; Muir et al. 2008). In coregonids, the oral part of the cycloid scales (Bagenal 1978) is used to estimate the relationship between scale radius and body length (Klein 1990), which can be used to calculate the size of fish in different age classes (Fisher and Pearcy 1990; Walker and Sutton 2016; Peterson et al. 2021).

Monitoring programs using net fisheries document the condition of captured fish, for example, age, length and weight at the time of capture. However, individuals are usually not caught equally across all age‐classes present, typically causing the juvenile age‐classes to be missing. Therefore, data sets providing information about long term fish growth across multiple age‐classes is seldom available.

Here we use digital scale images from a long‐term time series from a German pre‐alpine lake to analyze the growth history of age 3+ coregonids (i.e., individuals which have not yet completed their fourth year of life) over a time span of 22 years. No other age classes were included. Using this unique data set, we aim to test for potential changes in age‐related fish growth patterns and to assess associated effects of temperature and nutrient status.

## 2 Materials

Lake Starnberg is a large, monomictic pre‐alpine lake in southern Germany with a maximum depth of 127 m and a catchment area of 314.7 km^2^. Despite its alpine location, it lacks major alpine inflows and is mainly fed by small surface tributaries and groundwater springs (Riedmüller et al. 2022). Scales from 124 fish were analyzed, originating primarily from commercial catches, but also from routine monitoring programs conducted between 1998 and 2020. Fisheries regulations at Lake Starnberg are designed to avoid harvesting individuals that have not yet reached sexual maturity, and mesh sizes are adjusted accordingly. To ensure comparability and avoid age‐related bias, only fish aged 3+ were included in the analysis. This age group dominates the catch and is also most relevant from a fisheries management perspective, as these individuals are typically reproductively active and have contributed to population renewal.

Sampling was conducted between August and September using pelagic gillnets targeting adult Coregonus sp. in the open‐water zone. In the years 2000, 2005, and 2008, some individuals caught in July were included due to limited sample availability during the standard period.

**FIGURE 1:**
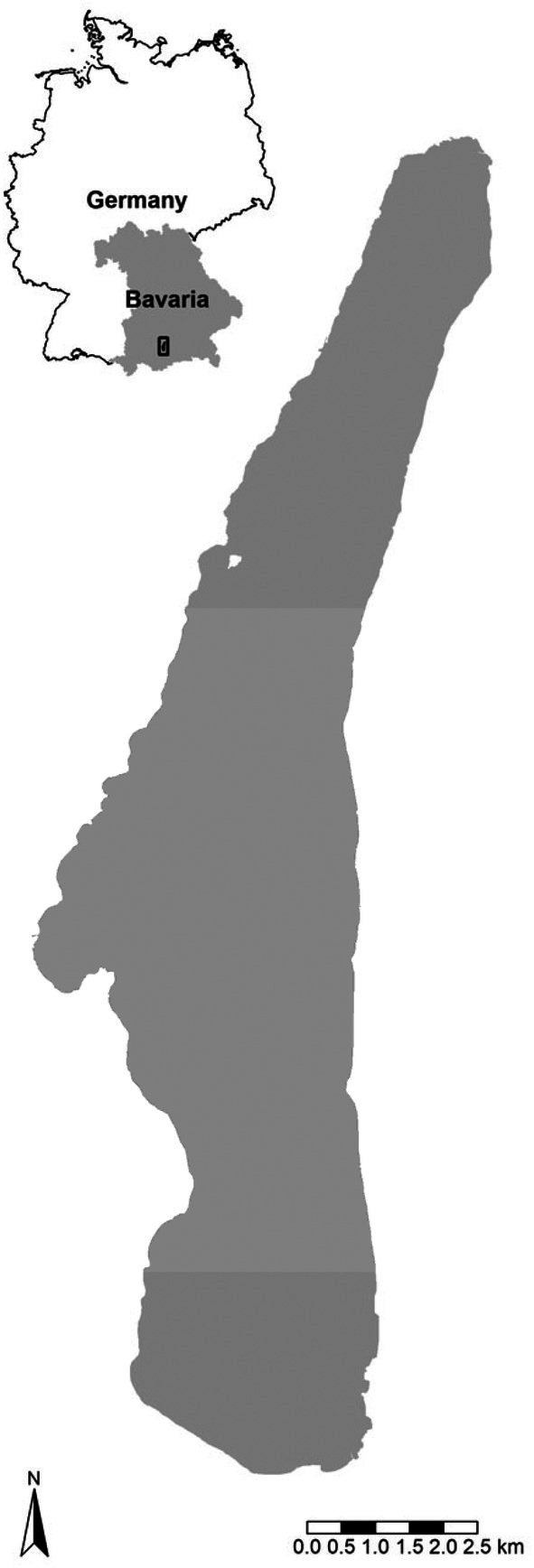
Map of Lake Starnberg, Germany.

Fish were captured through two methods: by commercial fisheries (using monofilament gillnets with mesh sizes of 40 mm from 1996 to 2013 and 36/38 mm from 2014 to 2020) and through scientific monitoring programs employing multimesh gillnets with panels of 44, 40, 35, 30, 25, and 20 mm (see Figure 1). To ensure consistency in size selectivity, only fish caught in mesh sizes ≥ 35 mm (MW 35) were included in this study. Sampled fish were measured to the nearest millimeter for fork length, weighed, and scales were collected from the area just above the anal fin, following Einsele (1943) and Lehtonen and Niemelä (1998). Scales were cleaned with dishwashing solution and the epidermis removed by careful brushing with an interdental brush. Fish age was independently verified by two experienced staff members using a binocular microscope. Only fish that were determined to be the same age by both operators were selected. This resulted in 5–9 fish per year and 3–10 scales per fish, with a total of 470 scales being retained for further processing and analysis (Table 1). The scales were fastened between a slide and one half of a slide frame and digitized with a slide scanner (reflecta CrystalScan 7200) at 3600 dpi. The scanned images were processed using Fidji software (Schindelin et al. 2012), using the convolve, make binary, and skeletonize filters. Radius length was measured from the scale center to the individual annuli (largest radius of the oral part of the scale). Subsequently, four measurements were taken from each scale, starting from the center to the first annuli (r1) to the second annuli (r2) to the third annuli (r3) and to the edge (rt). To determine the increment of each growth year, the smaller measurement was subtracted from the next larger measurement, for example, r2 − r1 = increment r2. Distance was measured in pixels or pixels per mm2. For temperature and phosphorus data (Bavarian Environment Agency (LfU Bayern) 2024), mean values for each year were calculated for February–June. These measurements were taken from the water surface, above the deepest point of the lake. The number of observations ranged between 1 and 8 per year taken from February to June. Exact numbers of observations are shown in Figure 4.

**TABLE 1:** Annual sample sizes for coregonid fish and scales from Lake Starnberg (1998–2020).

Year	Fish (*n*)	Scales (*n*)
1998	5	15
1999	5	15
2000	5	15
2001	9	47
2002	5	15
2003	5	15
2004	5	15
2005	5	15
2006	5	21
2007	5	15
2008	5	15
2009	5	17
2010	6	28
2011	5	15
2012	5	15
2013	5	19
2014	5	15
2015	5	30
2016	5	15
2017	5	15
2018	5	15
2019	8	24
2020	6	53

All analyses were performed using R statistical Software (R Development Core Team 2023). Simple linear regression was used to test for correlations between scale radius length and fish length, as well as between nutrients and year. Using the R package nlme (Pinheiro et al. 2023), nested linear mixed effect models (LMMs) were applied to test for effects of year, phosphorus, temperature, and age class on scale radius length. A two‐step procedure was chosen to reflect the individual questions of (i) possible age‐class‐dependent growth change by year and (ii) the role that temperature and phosphorus may play if such change is found to be present. For the latter analysis, only one model was specified according to the question being asked, rather than opting for a full model with multiple higher order interactions, which, even after model simplification, may be difficult to interpret biologically. An individual identifier was given to each scale and each fish, which were used as random factors in the mixed effect model to account for the hierarchy of radius measurement *i*, nested in scale *j*, nested in fish *k*. This resulted in the following two models, (i) excluding and (ii) including environmental effects:

- radius_length ∼ year_zero + age_class + year_zero:age_class + (1∣fish_id/scale_id)
- radius_length ∼ year_zero + temp + phos + age_class + temp:age_class + phos:age_class + year_zero:age_class + (1∣fish_id/scale_id)

Effects of the interaction terms specified in the LMMs were further investigated, using tools provided by the emmeans R package (Lenth 2024). Prior to fitting any models, the data was checked for outliers, collinearity, possible interactions and non‐linear patterns as outlined in Zuur, Ieno, and Elphick (2009). Model residuals were checked for homogeneity of variance, non‐linear patterns and independence according to Zuur et al. (2009) and Zuur and Ieno (2016). Residuals were non‐normally distributed, but as regression techniques are generally robust against deviations from normality (Knief and Forstmeier 2021) no data transformations were performed. Otherwise, raw data and model residuals were found to be in accordance with model assumptions, except for heteroscedasticity detected in the residuals of the linear regression of temperature and year. Here, robust standard errors from the sandwich R package were used to allow for violation of the homogeneity of variance assumption in the linear model (Zeileis et al. 2020; Zeileis 2004). To avoid common pitfalls associated with stepwise model selection, for example, bias in parameter estimation and an inappropriate reliance on a single best model (Whittingham et al. 2006), no model simplification was conducted. We report both estimated coefficients and overall significance tests. Coefficient estimates, standard errors, and *t*‐values provide information on effect magnitude and direction. Overall significance of each variable is assessed using Type III ANOVA tests, which account for all other terms in the model and are appropriate for unbalanced designs with interactions.

## 3 Results

**FIGURE 2:**
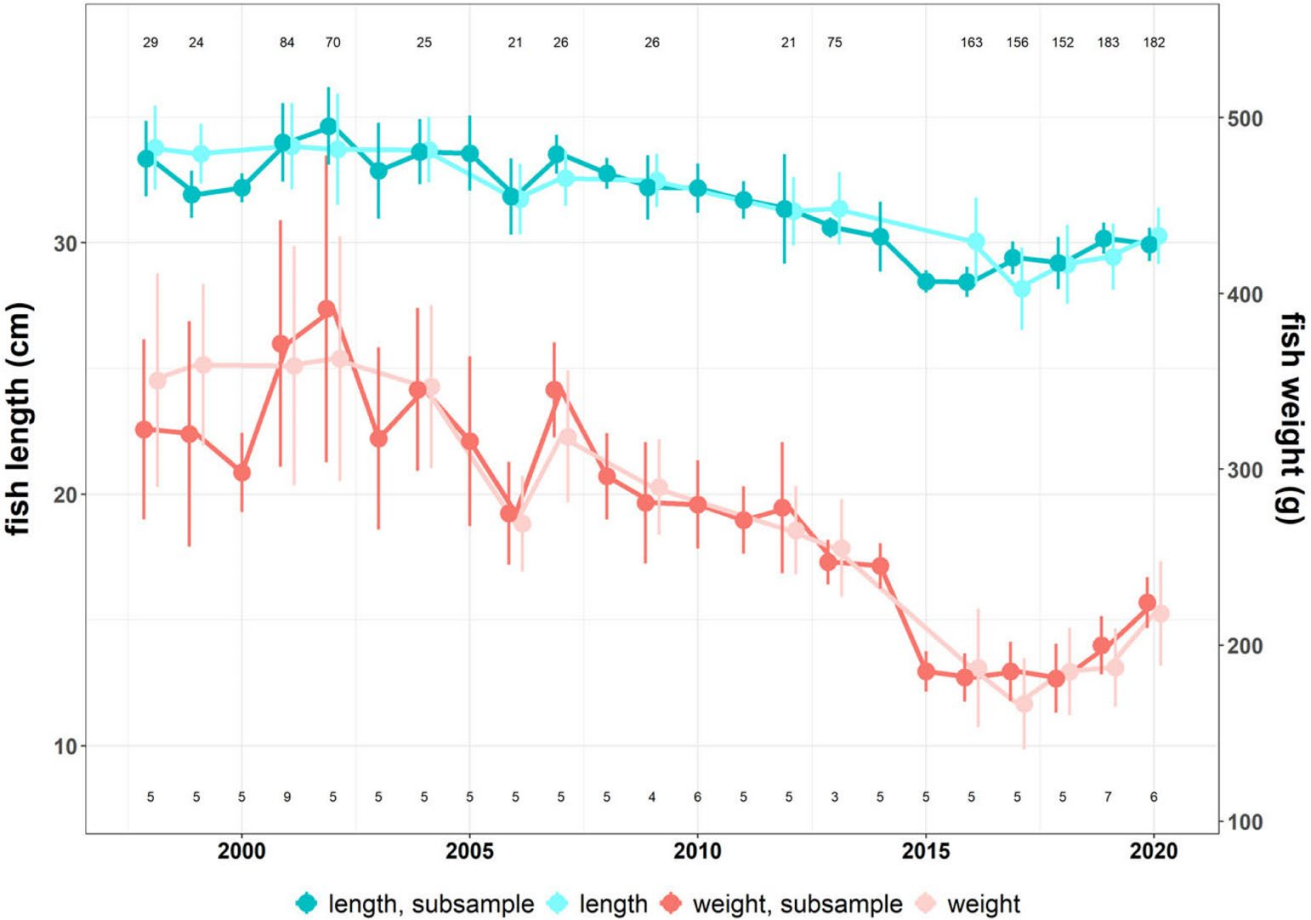
Coregonid length and weight with standard deviation from Lake Starnberg caught between 1998 and 2020 (August–October). Darker colors show the subsample of fish included in the scale analysis, while lighter colors incorporate all individuals sampled during this period. Numbers at the top and bottom of the plot give the sample size for length and weight of all individuals caught per year, respectively.

Coregonids from Lake Starnberg showed a decrease in both length and particularly weight between 1998 and 2015 (Figure 2). After that period, length and size appear to stabilize and increase until the end of the available data set in 2020 (Figure 2). Mean fish length per year for the analyzed subsample ranged between 28.44 and 34.64 cm (2016 and 2002, respectively), while mean fish weight per year varied between 181.2 and 391.2 g (2018 and 2002, respectively). There was no significant difference in length or weight between all caught fish and the subsample used for the scale analysis (two sample *t*‐test: *t* = 0.08 and 0.11, df = 28.64 and 26.5, both *p* > 0.05, respectively).

**FIGURE 3:**
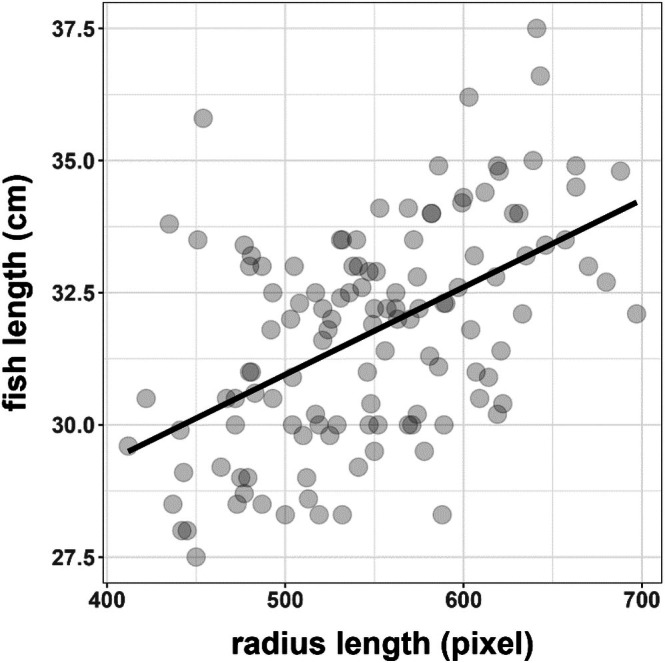
Regression between fish length (measured as fork length) and radius length of all examined coregonids from Lake Starnberg (1998–2020).

Regression analysis of fish and radius length (Figure 3) shows a significant positive correlation (*y* = 0.017*x* + 22.686, *F*_1,121_ = 42.46, *r* ^2^ = 0.25, *p* < 0.01).

**FIGURE 4:**
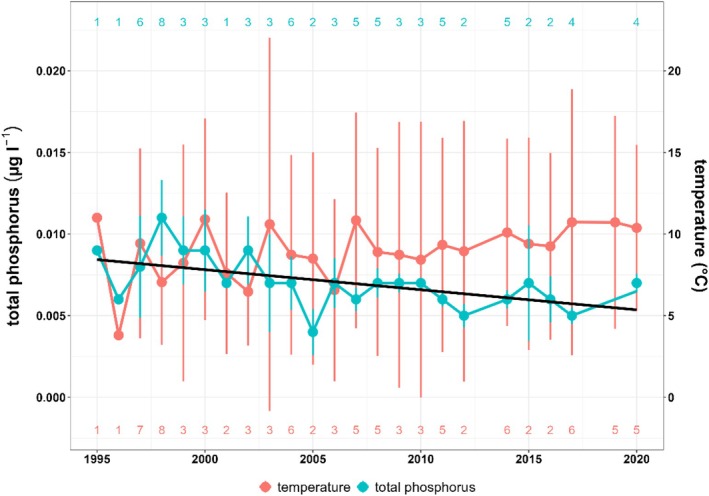
Mean values and standard deviation of total phosphorus and temperature between 1995 and 2020 from Lake Starnberg, Germany. Mean values per year encompass data from February to June. Number of observations are given at the top and bottom of the graph. All observations taken from water surface. The black regression line shows the significant relationship between total phosphorus and year.

After accounting for heteroscedasticity in the model residuals by computing robust standard errors, no significant change of water temperature by year was found (*β* = 0.096 ± 0.058 SE, *t* = 1.66, *p* = 0.112; Figure 4). Total phosphorus, however, significantly decreased during the observed time period (*y* = −0.000123*x* + 0.2538, *F*_1,21_ = 9.82, *r* ^2^ = 0.32, *p* = 0.005; Figure 4). Mean temperatures per year ranged between 3.8°C and 11°C 996 and 1995, respectively, while mean phosphorus readings per year varied between 0.004 and 0.011 μg L^−1^ (1998 and 2005, respectively, Figure 4).

**FIGURE 5:**
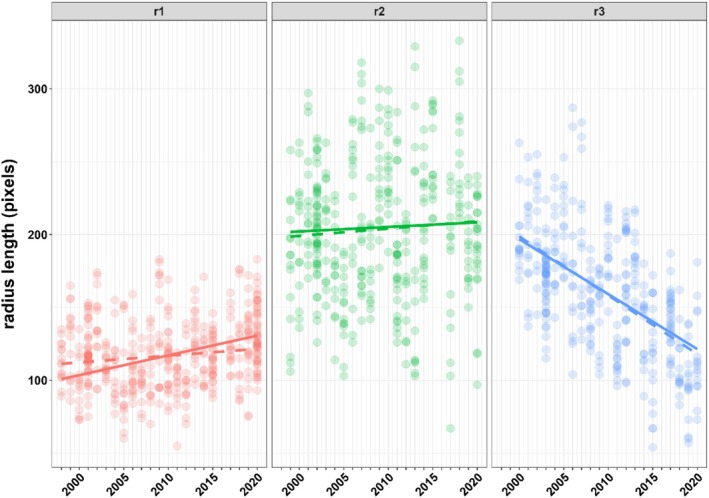
Model fit for change in scale radius length by year and age class, based on linear mixed effect models for (i) radius length as a function of year, age class and subsequent interaction (dashed lines), and (ii) radius length as a function of year, temperature, phosphorus and age class, as well as interactions between temperature and phosphorus with age class. Each point represents a single scale measurement. Multiple scales were measured per individual fish; the mixed‐effects model explicitly accounts for this repeated‐measures structure by including random effects for both fish and scale.

Linear mixed effect modeling of scale growth as a function of year and age class revealed a significant interaction between these terms, that is, change in scale growth by year differs by age class (Table 2). Here, age class 3 shows a strong significant decrease in scale growth during the study period, while age classes 1 and 2 do not exhibit any significant change by year (Table 3, Figure 5). Following the principle of marginality, it is difficult to interpret main effects in the presence of a significant interaction (Venables 1998). Therefore, the main effects temperature and age class are not further discussed.

**TABLE 2:** ANOVA results (type III sum of squares) from linear mixed effects modeling of year, age class, and subsequent interaction term on coregonid scale growth.

Variable	*χ* ^2^	df	Pr(>*χ* ^2^)
(Intercept)	688.48	1	0.0000
Year	2.34	1	0.1258
Age class	489.15	2	0.0000
Year × age class	172.50	2	0.0000

**TABLE 3:** Estimated marginal means for the interaction term between year and age class based on linear mixed effects modeling of year, age class and subsequent interaction term on coregonid scale growth.

Age class	Coefficient	SE	df	Lower CL	Upper CL	*t* ratio	*p*
r1	0.46	0.30	730	−0.13	1.06	1.53	0.126
r2	0.51	0.34	730	−0.15	1.16	1.51	0.133
r3	−4.03	0.36	730	−4.74	−3.33	−11.21	0.000

In the second analysis, temperature and phosphorus, as well as their interactions with age class were added to the model. In this case, all terms were found to be significant, except for the interaction between phosphorus and age class (Table 4). Differences in scale growth by year between age classes were similar to results from the previous model, however, age class 1 now shows a significant increase in scale growth by year (Figure 5, Table 5). This suggests that environmental fluctuations were masking underlying temporal trends in juvenile growth, emphasizing the importance of including these variables in the analysis. The significant interaction between temperature and year indicates that the effect of temperature on growth differs by age class (Table 6). Here, we find a negative slope for age‐classes 1 and 3, but a positive slope for age‐class 2 (Table 6). Variance component analysis of the random term structure shows that in both models there is virtually no unexplained variation between scales from the same fish (Tables 7 and 8). Both models attribute approximately 20% of the variance to differences between individual fish (Tables 7 and 8), while the remaining ~80% is residual variance not captured by the model (Tables 7 and 8).

**TABLE 4:** ANOVA results (type III sum of squares) from linear mixed effects modeling of year, temperature, phosphorus, and age class, as well as interactions between temperature and phosphorus with age class on coregonid scale growth.

Variable	*χ* ^2^	df	Pr(>*χ* ^2^)
(Intercept)	33.33	1	0.0000
Year	11.18	1	0.0008
Temperature	4.31	1	0.0379
Phosphorus	7.90	1	0.0050
Age class	26.51	2	0.0000
Temperature × age class	18.04	2	0.0001
Phosphorus × age class	1.95	2	0.3767
Year × age class	92.64	2	0.0000

**TABLE 5:** Estimated marginal means for the interaction term between year and age class based on linear mixed effects modeling of year, temperature, phosphorus and age class, as well as interactions between temperature and phosphorus with age class on coregonid scale growth.

Age class	Coefficient	SE	df	Lower CL	Upper CL	*t* ratio	*p*
r1	1.35	0.41	724	0.56	2.15	3.34	0.001
r2	0.31	0.46	724	−0.59	1.21	0.68	0.498
r3	−3.77	0.48	724	−4.70	−2.84	−7.94	0.000

**TABLE 6:** Estimated marginal means for the interaction term between temperature and age class based on linear mixed effects modeling of year, temperature, phosphorus, and age class, as well as interactions between temperature and phosphorus with age class on coregonid scale growth.

Age class	Coefficient	SE	df	Lower CL	Upper CL	*t* ratio	*p*
r1	−2.50	1.20	724	−4.86	−0.14	−2.08	0.038
r2	5.14	1.43	724	2.34	7.95	3.60	0.000
r3	−2.89	1.52	724	−5.88	0.10	−1.90	0.058

**TABLE 7:** Variance components table for the random term structure of the linear mixed effects model of year, age class, and subsequent interaction term on coregonid scale growth.

Level	Variance	SD	% of Total
Between fish	229.735100	15.157015	17.8000
Between scales (within fish)	0.000003	0.001737	0.0000
Residual	1061.014000	32.573210	82.2000
Total	1290.749103	35.926997	100.0000

**TABLE 8:** Variance components table for the random term structure of the linear mixed effects model of year, temperature, phosphorus and age class, as well as interactions between temperature and phosphorus with age class on coregonid scale growth.

Level	Variance	SD	% of Total
Between fish	253.749600	15.930000	19.800
Between scales (within fish)	0.000004	0.001890	0.000
Residual	1029.982000	32.090000	80.200
Total	1283.731604	35.829200	100.000

## 4 Discussion

Our long‐term analyses of growth patterns showed a striking pattern: while growth significantly increased over the 22‐year sampling period in the first age class, there was a strong significant decline for age class 3. This is in line with life history theory, which predicts that the costs of accelerated growth become apparent after the onset of maturity, as age 3+ whitefish in Lake Starnberg year have already reproduced. Such theoretical predictions are often difficult to validate with experimental or field data. In experiments, the necessary time scale of observations makes testing such predictions difficult except for fast‐growing organisms such as microbes. In the field, maintaining monitoring programs that span a sufficiently long time period to enable following the temporal growth of individual vertebrates is seldom achieved. Additionally, confounding environmental variables sometimes make it difficult to estimate whether shifts in growth patterns are triggered by fitness‐related trade‐offs between investment in juvenile and adult growth or driven by fluctuating environmental parameters such as temperature or resource availability.

In our study, we use spring/early summer temperatures for the most pronounced growth season and the available amount of phosphorus in the lake as a proxy for resources. Lake Starnberg is a deep oligotrophic prealpine lake, typically highly P limited, and the amount of P in the system (total P) is a well‐established proxy for plankton and fish growth (Lyche‐Solheim et al. 2013; Ochocka and Pasztaleniec 2016). By using scale analyses, we are able to assemble a 22‐year‐long data set, which allows the reconstruction of the growth history of individual fish (Figures 2 and 3). This cannot be done based on catch data alone, as information about size and weight gives no indication of past growth.

Our study uses a small number of fish samples per year (5–9), which increases variability and may reduce power to detect subtle effects. However, the 22‐year time series and the alignment of observed trends with life‐history theory support the robustness of our findings. Nonetheless, the low sample size introduces some uncertainty, and results should be interpreted with caution.

### 4.1 Limitations and Constraints of Scale Analysis as a Proxy for Growth

Studies show that age determination based on otoliths or other hard structures can sometimes provide more accurate results than scale analysis (Mills et al. 2004; Muir et al. 2008). Further, unexpected nutrient inputs from, for example, flooding or heavy rain events, or highly variable temperatures can potentially lead to misinterpretation of scale patterns (Campana 2001; Muir et al. 2008), especially in the posterior regions (Chilton and Beamish 1982). In contrast to the use of other hard structures, however, scale analysis allows age monitoring that is non‐invasive, as fish can be released after the scales have been removed. In addition, scale analysis provides the opportunity to gather data from fish from commercial fishermen, without degrading market value. Further, circuli growth around the scale center and scale radius both correlate well with body length (Chilton and Beamish 1982; Fisher and Pearcy 1990; Beakes et al. 2014; Thomas et al. 2019; Peterson et al. 2021) and appear to be independent of environmental factors (Thomas et al. 2019). A study by Reckahn (1986) shows that the scale diameter of coregonids is well suited for correlations with body weight. While our analysis focuses on the relationship between scale radius and fish length (Figure 3), these findings are consistent with the general utility of scale measurements as proxies for somatic growth. A further limitation of retrospective scale analysis is that it inherently reflects only the growth trajectories of fish that survived to the age of capture. Individuals that may have died earlier due to size‐ or growth‐selective mortality—particularly during the first years of life—are not represented in the dataset. This introduces a potential survivorship bias, especially during early growth stages (r1–r2), where such selective pressures are often strongest. While the present study focuses on age‐3+ individuals relevant for sustainable fisheries management, the influence of early‐life selection must be considered when interpreting growth patterns based on surviving fish.

### 4.2 Nutrient Supply as a Reason for Observed Growth Patterns

Many studies have shown that changes in nutrient supply have effects on fish growth (Downing et al. 1990; Mueller and Bia 1998), suggesting nutrient‐related changes in the quality and quantity of food as a likely cause (Straile 2000; Jeppesen et al. 2002; Müller et al. 2007). Higher food availability leads to increased somatic growth and allows more energy for reproductive processes (Thomas and Eckmann 2007). While zooplankton surveys at Lake Starnberg show that nutrient abundance was at a minimum in 2017 (Vogelmann et al. 2022), subsequent monitoring results up until 2022 indicate that the quantity and quality, that is, a higher proportion of daphnids, of the nutrient supply has improved (Institute for Fisheries Bavaria) (Figure 2). Similar results were obtained by Lumb et al. (2007), who showed that fish growth is regulated by zooplankton quality and quantity. Average fish weight from corresponding catch statistics (Figure 2) also shows an increase after 2017 in the age classes 1, 2, and 3+ compared to previous years. In contrast to our own results, which show a marked decline in growth over time for 3+ coregonids, Klein (1990) found that 3+ and older age classes still showed significant increases in growth for these fish at the neighboring Lake Wörthsee. In addition, his results also illustrate that growth in length remains constant, especially in the first year of life. Similarly, at Lake Lucerne, decreasing phosphorus concentration did not reduce the growth of coregonid age classes 1 to 3 over a period of almost 20 years (Mueller and Bia 1998). Our data also revealed that phosphorus was not a single significant driver of the observed growth patterns over the last 22 years. Phosphorus concentrations in the lake declined, but growth rates of first age classes still increased.

### 4.3 Temperature Effects

Long‐term studies by Reckahn (1986) showed that temperature was significantly correlated with coregonid weight. An increase in temperature normally leads to an increased metabolic rate in fish. As a result, a higher food consumption is necessary to maintain body size (Wootton 1998). With maximum food availability, optimal temperatures, longer growing seasons, and higher average trophic levels, increased growth may occur (Viljanen 1988; Plumb and Moffitt 2015). In contrast, when temperatures are too high, the opposite occurs, and growth rates decrease (Dabrowski 1985). The growth analyses at Lake Starnberg (Figure 5) show that the temperature (Figure 4) in Lake Starnberg had a significant interaction with age class. Our results suggest that the observed temperature variation in Lake Starnberg (Figure 4) did not alone contribute to shifts in growth rates (Figure 5). Our findings are consistent with Thomas et al. (2019), who argued that variation in circuli formation and spacing reflects individual growth patterns more accurately than external drivers like temperature, highlighting the complexity of growth regulation in fish.

### 4.4 Eco‐Evolutionary Effects on Coregonid Growth Patterns

Our study suggests that the scale width or the area of individual scales of fishes begins to vary along the observed time scale only after the second or third year of life. For comparison, the maturity of whitefish in Lake Lucerne largely begins at the end of the second year. By the end of the third year of life, all whitefish are mature (Mueller and Bia 1998). Studies by Gassner et al. (1998) also show that a majority of whitefish become mature in their second and third years of life. A possible decrease in growth in the third year of life of coregonids in Lake Starnberg could also be due to the use of energy resources in gonad production. Studies in other fish species (Heino 1999) show that as long as the fish is immature, its energy is invested in somatic growth. Similarly, Campana (2001) points out that gamete production or reproductive processes lead to a substantial decrease in growth rate. A study by Engelhard et al. (2003) on herring shows that width measurements of scale annual rings can be used to interpret sexual maturity. Our results suggest that especially in the third year (Figure 4), where radius length decreases significantly over time, substantial energy is invested in gonad production. Energy, primarily in the form of fat, is diverted from muscle to the gonads during this time (Dabrowski 1982). Several environmental factors can potentially affect the energy allocation of fish growth during vitellogenesis (Dabrowski 1985). Our results could reflect an earlier maturation of whitefish during the 22 years observational period, accompanied by an increased juvenile growth.

While some studies (e.g., van der Sleen et al. 2018) have analyzed juvenile and adult growth separately due to distinct ecological contexts, we chose to model all age classes together, as our fish were sampled from the same environment and direct comparison between age classes is biologically justified. Our approach, which includes age class as a factor and tests for interactions with environmental variables, allows us to capture life‐history trade‐offs without requiring separate analyses.

Another factor that can influence fish growth is population density, which may lead to intraspecific competition at high densities (Lorenzen and Enberg 2002; Götz 2007; Schmidt and Schubert 2018). Hydroacoustic surveys at Lake Starnberg in 2007 and 2017 resulted in density estimates of 28 kg/ha and 355 Ind/ha, and 40 kg/ha and 578 Ind/ha, respectively. These values do not indicate strong intraspecific competition.

Another reason for the decrease in size of 3+ fish could be the result of size‐selective mortality, for example, by fisheries gear or net mesh size selection. Most fishing methods have direct effects on fish size and age selection (Heino et al. 2015). Very early on, Rutter (1903) noted evolutionary changes due to selective fishing. Fishery‐induced selection also occurs due to the protection of smaller individuals, the capture of specific species, or for economic efficiency (Andersen et al. 2012; Yin et al. 2024). Highly selective removal of large individuals can lead evolutionarily to smaller body size as well as earlier sexual maturation at smaller body size (Van Wijk et al. 2013). Hence, body size in general and age‐specific size (Ricker 1981) may decrease due to fisheries‐induced evolutionary changes in population structure. On the other hand, the opposite can also occur because of fishing exploitation. Reducing population density makes more resources available to individuals and can thus accelerate the growth of single individuals (Law 2000). In their studies of so‐called “Blaufelchen” ( *Coregonus wartmanni* ) at Lake Constance, Thomas and Eckmann (2007) show that the age and size of female individuals are related to fishing pressure and size‐selective fishing, respectively. In their studies, they conclude that size‐selective fishing has caused “Blaufelchen”‐ populations to mature earlier. Investing energy into earlier reproduction rather than somatic growth seems to be the more successful response to fishing pressure. Hence, life history theory predicts a simultaneous increase of growth in early life during the juvenile phase, such as visible in our data.

Fishing yield and body size of 3+ coregonids in Lake Starnberg have strongly declined in recent years. If changes in environmental drivers such as temperature or nutrient supply were the main reason for the observed declines, a decrease in body size of whitefish during the first and second year of life would also be expected. However, it is important to note that strong size‐selective mortality, particularly during early life stages, may mask such trends, as slower‐growing or smaller individuals are less likely to survive. However, we observe the opposite pattern, where growth increases for the first age class, while growth decreases for the mature individuals in age class 3. Such patterns are typical for life history trade‐offs between investment in reproduction and growth. Earlier reproduction results in the decline of somatic growth as soon as allocation of resources to reproduction starts but is often accompanied by increased juvenile growth. Our unique data set, spanning 22 years and multiple age classes of whitefish, enabled us to show a clear life history response of a fish population, as expected by evolutionary theory. To our knowledge, this is one of the few studies that have demonstrated a trade‐off between growth in early and late life history stages in a natural fish population, observed over more than two decades.

## Author Contributions

**Christian Vogelmann:** conceptualization (equal), data curation (equal), investigation (equal), methodology (equal), writing – original draft (equal), writing – review and editing (equal). **Maxim Teichert:** formal analysis (equal), methodology (equal), visualization (equal), writing – original draft (equal), writing – review and editing (equal). **Michael Schubert:** conceptualization (equal), methodology (equal), writing – review and editing (equal). **Niels Dingemanse:** formal analysis (equal), writing – review and editing (equal). **Herwig Stibor:** conceptualization (equal), supervision (equal), writing – original draft (equal), writing – review and editing (equal).

## Conflicts of Interest

The authors declare no conflicts of interest.

## Supporting information


Data S1.

## Data Availability

The data supporting the findings of this study are openly available on Figshare at https://doi.org/10.6084/m9.figshare.28601408.v1. Additionally, the analysis code used in this study can be accessed on GitHub at https://github.com/maxim‐teichert/scale‐growth‐analysis.git.
